# [^18^F]FDG-6-P as a novel *in vivo* tool for imaging staphylococcal infections

**DOI:** 10.1186/s13550-015-0095-1

**Published:** 2015-03-19

**Authors:** Bethany Mills, Ramla O Awais, Jeni Luckett, Dave Turton, Paul Williams, Alan C Perkins, Philip J Hill

**Affiliations:** School of Life Sciences, Centre for Biomolecular Sciences, University of Nottingham, University Boulevard, Nottingham, NG7 2RD UK; School of Medicine, University of Nottingham, Nottingham, NG7 2RD UK; PETNET Solutions, Nottingham City Hospital, Hucknall Road, Nottingham, NG5 1 PB UK; School of Biosciences, University of Nottingham, Sutton Bonington Campus, Sutton Bonington, LE12 5RD UK

**Keywords:** Pre-clinical, NanoPET-CT imaging, *S. aureus*, Infection diagnosis

## Abstract

**Background:**

Management of infection is a major clinical problem. *Staphylococcus aureus* is a Gram-positive bacterium which colonises approximately one third of the adult human population. Staphylococcal infections can be life-threatening and are frequently complicated by multi-antibiotic resistant strains including methicillin-resistant *S. aureus* (MRSA). Fluorodeoxyglucose ([^18^F]FDG) imaging has been used to identify infection sites; however, it is unable to distinguish between sterile inflammation and bacterial load. We have modified [^18^F]FDG by phosphorylation, producing [^18^F]FDG-6-P to facilitate specific uptake and accumulation by *S. aureus* through hexose phosphate transporters, which are not present in mammalian cell membranes. This approach leads to the specific uptake of the radiopharmaceutical into the bacteria and not the sites of sterile inflammation.

**Methods:**

[^18^F]FDG-6-P was synthesised from [^18^F]FDG. Yield, purity and stability were confirmed by RP-HPLC and iTLC. The specificity of [^18^F]FDG-6-P for the bacterial universal hexose phosphate transporter (UHPT) was confirmed with S. *aureus* and mammalian cell assays *in vitro*. Whole body biodistribution and accumulation of [^18^F]FDG-6-P at the sites of bioluminescent staphylococcal infection were established in a murine foreign body infection model.

**Results:**

*In vitro* validation assays demonstrated that [^18^F]FDG-6-P was stable and specifically transported into *S. aureus* but not mammalian cells. [^18^F]FDG-6-P was elevated at the sites of *S. aureus* infection *in vivo* compared to uninfected controls; however, the increase in signal was not significant and unexpectedly, the whole-body biodistribution of [^18^F]FDG-6-P was similar to that of [^18^F]FDG.

**Conclusions:**

Despite conclusive *in vitro* validation, [^18^F]FDG-6-P did not behave as predicted *in vivo*. However at the site of known infection, [^18^F]FDG-6-P levels were elevated compared with uninfected controls, providing a higher signal-to-noise ratio. The bacterial UHPT can transport hexose phosphates other than glucose, and therefore alternative sugars may show differential biodistribution and provide a means for specific bacterial detection.

## Background

Nuclear imaging provides a platform for identifying sites of bacterial infection in the clinic more rapidly than traditional diagnostic microbiology laboratory methods allow. However, the clinical imaging agents currently employed for this purpose (such as radiolabelled leukocytes [1-4] or [^18^F]FDG (2-fluoro-2-deoxy-D-glucose) [5-9]) are non-specific and as such may accumulate at sites of sterile inflammation or other lesions, resulting in a high rate of false positive results [9-15]. Despite concerted efforts over the past decade to develop bacteria-specific probes for direct nuclear imaging of infection, [^18^F]FDG remains the primary radiopharmaceutical for positron emission tomography (PET) imaging [5-9]; however, a number of other radiopharmaceuticals are currently under pre-clinical development [16,17]. Selective uptake into bacteria in *in vivo* experimental murine infection models exploits the ability of bacterial cells to internalise alternative sugars to mammalian cells. This offers a strategy that could be exploited for imaging bacterial infection sites independent of host inflammatory responses.

*Staphylococcus aureus* colonises 30% of the adult population at any one time, and hospital-acquired methicillin-resistant *S. aureus* (MRSA) strains (HA-MRSA) are the most common multi-drug resistant (MDR) bacteria isolated from European hospitals [18-21]. It causes a wide range of infections including skin and soft tissue (such as impetigo or abscesses), deep bone and foreign body infections that can lead to sepsis and pneumonia [22]. The high rate of MDR bacterial infections has, in large part, been exacerbated by misdiagnosis, and therefore inappropriate treatment [20,21,23,24]. Moreover, community-acquired MRSA (CA-MRSA) infections are growing in prevalence, causing disease in otherwise healthy individuals [25-29]. Therefore, developing new radiopharmaceuticals that enable rapid diagnosis of staphylococcal infections is imperative.

To this end, we have exploited an alternative sugar transporter, the bacterial universal hexose phosphate transporter (UHPT) [30]. The UHPT is expressed by many different Gram-positive and Gram-negative bacterial species including *S. aureus* and is induced by extracellular glucose-6-phosphate. It is the sole mechanism by which sugar phosphates are internalised into the staphylococcal cell. Consequently, this defined route for staphylococcal transport of glucose-6-phosphate makes UHPT an ideal candidate for the delivery of targeted bacteria-specific probes. [^18^F]FDG and [^18^F]FDG-6-P are analogues of glucose, and it is reasonable to assume that [^18^F]FDG-6-P would be selectively transported into bacterial cells, which, in contrast to mammalian cells, can internalise sugar phosphates from their surrounding environment. This would lead to high signal-to-background ratio and enables specific diagnosis of infection, rather than sterile inflammation. Here we report on the evaluation of [^18^F]FDG-6-P as an imaging agent for *S. aureus* infection *in vivo* using pre-clinical PET-CT.

## Methods

### Ethics statement

All animal experiments were approved by the University of Nottingham Animal Welfare and Ethical Review Board and performed in accordance with the UK Home Office Licence rules, under Project Licence 40/3821. All injected volumes were in line with ASPA guidelines.

### Statistical analysis

Statistical analysis was performed using Prism 6 (GraphPad Software Inc., La Jolla, CA, USA). Where appropriate, analyses were performed by student's *t*-test (for *in vitro* analysis) and Mann-Whitney *U* test (for *in vivo* analysis). Unless otherwise stated, for *in vitro* data, error bars show standard error of the mean (SEM); for *in vivo* data, bars on graphs show data median.

### Synthesis and analysis of [^18^F]FDG-6-P

[^18^F]FDG-6-P was prepared from commercially available [^18^F]FDG (PETNET Solutions, Nottingham, UK) following a method previously described [31]. The purity of the [^18^F]FDG-6-P product was determined by reverse-phase high-performance liquid chromatography (RP-HPLC) on an Agilent 1260 Series LC (Agilent Technologies, Sta. Clara, CA, USA) connected to Flow-RAM™ sodium iodide detector (LabLogic Systems Limited, Sheffield, UK). A SphereClone SAX 5 μm 250 × 4.6 mm column (Phenomenex, Torrance, CA, USA) was used for RP-HPLC at a flow rate of 0.8 ml min^−1^ with UV detection at 295 nm, using an isocratic method with 20 mmol l^−1^ potassium phosphate (pH 7.2) as the mobile phase. Instant thin layer chromatography (iTLC) was carried out using reverse-phase plates (Merck F254 aluminium sheet silica plates, 1.0 × 7.0 cm; Merck & Co., Whitehouse Station, NJ, USA). Radiolabelling efficiency on iTLC strips was measured using a Bioscan radio-TLC Scanner (LabLogic, Sheffield, UK). To ensure the stability of [^18^F]FDG-6-P over time, 2 MBq of [^18^F]FDG-6-P was incubated at 37°C for up to 3 h in 1 ml water or for 1 h in 1 ml blood (extracted from mice and heparinised). The products were analysed by HPLC (water) or iTLC (blood).

### [^18^F]FDG-6-P uptake assays

*S. aureus* RN6390 and a UHPT-deficient strain *S. aureus* RN6390 *ΔuhpT* were grown overnight in tryptic soy broth (TSB, Oxoid Microbiology Products; Thermo Fisher Scientific, Basingstoke, Hampshire, UK) at 37°C, with shaking at 250 rpm. The overnight cultures were diluted to OD_600_ 0.1 and incubated until mid-exponential phase was reached. One millilitre of cells was harvested (1 × 10^8^ CFU), washed and resuspended in 1 ml RPMI 1640 (Life Technologies, Carlsbad, CA, USA).

Jurkat, AGS, THP1 and HL60 cell lines were grown in RPMI 1640 tissue culture medium supplemented with L-glutamine, 10% *v*/*v* foetal calf serum (FCS) and 1% *w*/*v* penicillin/streptomycin (P/S). The cells were grown in 75-cm^2^ flasks at 37°C and 5% CO_2_. The cells were maintained at 0.5 to 1 × 10^6^ cells ml^−1^. HL60 cells were supplemented with 1.3% *v*/*v* DMSO for 3 to 4 days followed by 24 h without DMSO prior to experimentation in order to increase expression of CD11b. AGS cells were seeded into 6-well plates (Corning Inc., Corning, NY, USA) prior to experimentation.

HIB-1B and 3 T3-L1 cell lines were grown in DMEM (Gibco; Life Technologies) tissue culture medium, supplemented with 10% *v*/*v* FCS, 1% *w*/*v* P/S and 1% *w*/*v* sodium pyruvate. The cells were grown in 75 cm^2^ flasks at 37°C and 5% CO_2_. The cells did not exceed 80% confluence. HIB-1B cell lines were seeded into 6-well plates and underwent differentiation by incubation with DMEM supplemented with 10% *v*/*v* FCS, 1% P/S, 1% sodium pyruvate, 3-isobutyl-L-methylxanthine (IBMX, 500 μM), dexamethasone (DEX, 250 nM), insulin (170 nM) and triiodo-L-thyronine (T3, 10 nM) for 48 h. The cells were then incubated with fresh DMEM supplemented with 2% *v*/*v* FCS, T3 (10 nM) and insulin (170 nM) every 48 h for 8 days. The 3 T3-L1 cells were seeded into 6-well plates and underwent differentiation by incubation with DMEM supplemented with 10% *v*/*v* FCS, 1% *w*/*v* P/S, 1% *w*/*v* sodium pyruvate, IBMX (500 μM), DEX (250 nM) and insulin (170 nM) for 48 h. The cells were then incubated in fresh DMEM supplemented with 10% *v*/*v* FCS, 1% P/S, 1% *w*/*v* sodium pyruvate and insulin (170 nM) every 48 h for 8 days.

For experimentation, non-adherent cell lines (HL60, THP1, Jurkat; 1 × 10^6^ cells ml^−1^) were harvested, washed and resuspended in 1 ml tissue culture medium. The adherent cell lines (AGS, HIB-1B and 3 T3-L1) were maintained in 6-well plates and were 80% confluent. Each cell type (bacterial or mammalian) and controls without cells were incubated with 2 MBq [^18^F]FDG or 2 MBq [^18^F]FDG-6-P for 1 h at 37°C. Bacteria and non-adherent mammalian cells were harvested by centrifugation (600 × *g*, 5 min) and washed three times by centrifugation. All supernatants were collected for each sample in scintillation vials. After washing, the cells were transferred into scintillation vials. Adherent cell lines were washed three times by replacing the well medium. Supernatants were collected into scintillation vials. The cells were removed from the wells by trypsin treatment and placed into scintillation vials. The scintillation vials for cells and supernatants were counted by gamma counter (1480 Automatic Gamma Counter, PerkinElmer Inc., Waltham, MA, USA), and the results were obtained as counts per min (cpm).

Results were normalised for controls (no cells) and by calculating the percentage of activity in the cell containing scintillation vials compared with the total counts (cells and supernatant combined). The percentage of activity associated with the cells was normalised so that [^18^F]FDG cell uptake was equivalent to 1. The samples were incubated in triplicate, and the data were presented from at least two independent repeats.

### Animal models and infection

Female BALB/c mice, 19 to 22 g (Charles River Laboratories, Tranent, UK) were used for all experimentation. All injections were performed using a 25-G insulin needle and syringe (Becton Dickinson and Co., Franklin Lakes, NJ, USA). Silicone 5-mm-long catheter sections (scale Fr 8, RUSCH Brilliant Pediatric, Teleflex Inc., Wayne, PA, USA) were implanted subcutaneously (sc) into the right flank of the mice as previously described [32,33], with the following modifications. Analgesia (carprofen (Rimadyl), 5 mg kg^−1^, Pfizer Inc., New York, NY, USA) was administered by sc injection 60 min prior to anaesthesia (inhalation of 2% isoflurane in oxygen). The incision site was shaved and cleansed with chlorhexidine gluconate (Hydrex Surgical Scrub) clear solution (Ecolab, Northwich, Cheshire, UK). After insertion of the catheter, the incision site was sealed with tissue adhesive (GLUture, Abbott Laboratories, Maidenhead, UK). The incision sites were healed for at least 7 days prior to inoculation with bacteria.

The bioluminescent (BL) *S. aureus* strain Xen29 (1 × 10^7^ CFU) in 50 μl saline (*n* = 6) or saline only (*n* = 5) was administered directly into the catheter lumen. Mice were imaged optically using an IVIS Spectrum (PerkinElmer) to confirm the presence of BL bacteria. The images were captured for 30 s with small (4 × 4) binning. Optical data was processed with Living Image 3.2 software (Caliper Life Sciences, Hopkinton, MA, USA). All mice were maintained under anaesthesia by inhalation of 2% *v*/*v* isoflurane in oxygen for the duration of imaging (optical or nuclear).

### NanoScan PET-CT imaging

At 24 h post bacterial inoculation, mice were injected with 10 MBq [^18^F]FDG (*n* = 3 infected, *n* = 3 uninfected) or [^18^F]FDG-6-P (*n* = 3 infected, *n* = 2 uninfected) by intraperitoneal (ip) injection. All scans were carried out 1 h later using a nanoScan PET-CT (Mediso Medical Imaging Systems, Budapest, Hungary) small animal scanner with the following parameters: CT: 1 field of view (FOV), maximum FOV, full scan, 720 projections; tube 35 kVP, 170 ms exposure, 1:1 binning. PET: coincidence 1:5, scan time 20 min, packet timestamp. Scanning and subsequent reconstruction were carried out using Nucline software (Mediso). Reconstruction parameters are as follows: 3D, whole body, non-dynamic with 1:3 binning.

### NanoScan PET-CT data analysis

All image analysis was performed with VivoQuant software (inviCRO LLC, Boston, MA, USA). All data were normalised for min/max counts based on exact radiopharmaceutical injection dose; radiopharmaceutical signal from bladders was masked and all images were scaled equally. 3D regions of interest (ROIs) were drawn manually or using integrated thresholding tools (global) where appropriate. Standardised uptake values (SUVs) were calculated for each ROI using the following formula: ROI concentration (MBq mm^−3^)/(Injected dose (MBq)/Mouse weight (kg)). To determine the infected to uninfected (I/UI) ratio of infected vs. uninfected catheters, the mean SUV for the infected mouse catheter was divided by the mean catheter SUV of the uninfected mouse.

## Results

### Synthesis and stability of [^18^F]FDG-6-P

**Figure 1:**
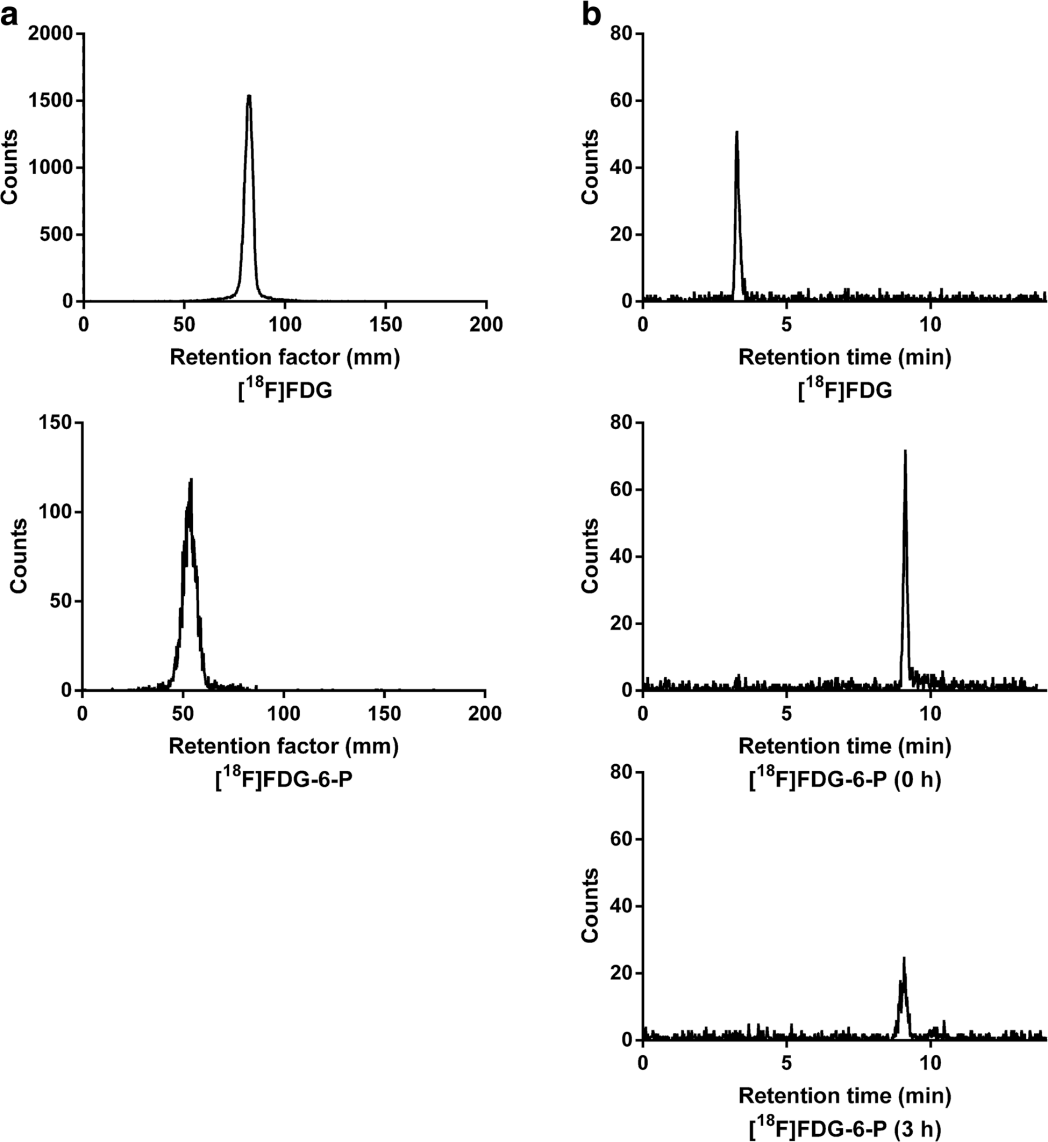
**Determination of the purity of [** ^**18**^ **F]FDG-6-P.** By **(a)** iTLC at 0 h and **(b)** RP-HPLC after incubation of the radiopharmaceutical in water for 3 h. The retention profiles of [^18^F]FDG-6-P were easily resolved from that of [^18^F]FDG, the most likely breakdown product of [^18^F]FDG-6-P. No changes in the [^18^F]FDG-6-P peak or retention time were observed.

[^18^F]FDG-6-P was synthesised from [^18^F]FDG as previously described [31]. The [^18^F]FDG-6-P product was analysed by iTLC (Figure 1a) and RP-HPLC (Figure 1b) in order to confirm yield and determine that the [^18^F]FDG-6-P peak could be distinguished from that of [^18^F]FDG by both methods. Analysis by HPLC determined that the retention time of [^18^F]FDG-6-P was approximately 8 min and that of [^18^F]FDG was approximately 3 min. The retention factors (Rf) of [^18^F]FDG-6-P and [^18^F]FDG measured by iTLC were 0.27 and 0.41 respectively. The stability of [^18^F]FDG-6-P over time was confirmed by HPLC analysis after incubation of the radiopharmaceutical in water for 3 h (Figure 1b).

### UHPT is required for [^18^F]FDG-6-P uptake

**Figure 2:**
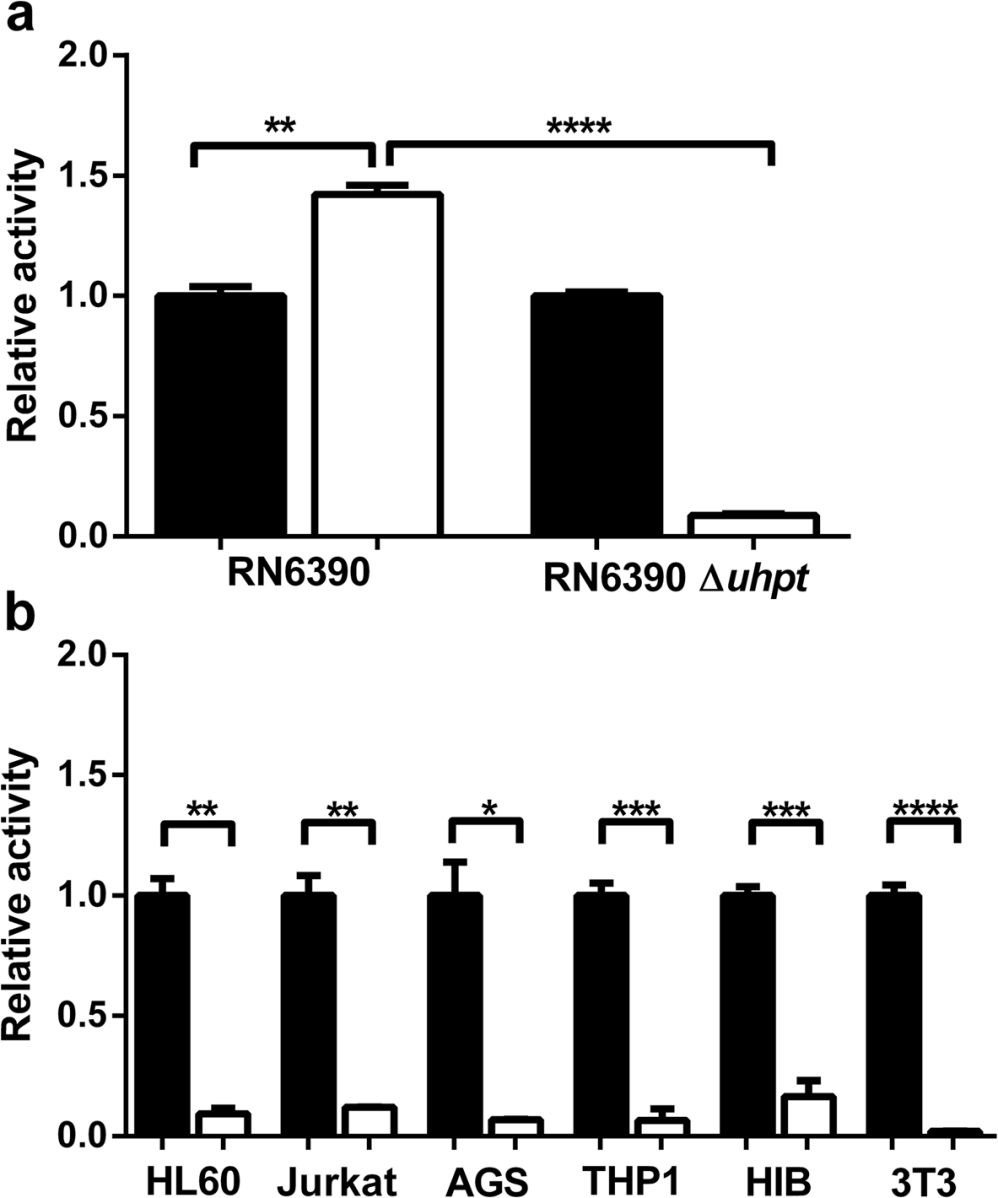
**Uptake of [** ^**18**^ **F]FDG-6-P and [** ^**18**^ **F]FDG by** ***S. aureus*** **and mammalian cells. (a)** *S. aureus* RN6390 and the isogenic UHPT-deficient mutant were incubated with either [^18^F]FDG or [^18^F]FDG-6-P. The activity associated with each of the strains after incubation with [^18^F]FDG (black) or [^18^F]FDG-6-P (white) was measured using a gamma counter. Counts were normalised to remove ‘no cell’ control counts and to compare [^18^F]FDG-6-P uptake with [^18^F]FDG uptake. Error bars show SEM. **P = 0.0015; ****P < 0.0001. Data were collected from three independent repeats. **(b)** The activity associated with mammalian cell lines HL60, Jurkat, AGS, THP1, HIB-1B and 3 T3-L1 after incubation with [^18^F]FDG (black) or [^18^F]FDG-6-P (white) was measured using a gamma counter. The counts were normalised to compare [^18^F]FDG-6-P uptake with [^18^F]FDG uptake. Error bars show SEM. *P < 0.05; **P < 0.01; ***P < 0.001; ****P < 0.0001. Differences in the means of cells incubated with [^18^F]FDG-6-P were not significant.

Despite [^18^F]FDG-6-P being a homologue of glucose-6-phosphate, it was not known whether this radiopharmaceutical would be transported by the staphylococcal UHPT. *S. aureus* RN6390 and UHPT-deficient *S. aureus* RN6390 *ΔuhpT* were incubated with 2 MBq of [^18^F]FDG or [^18^F]FDG-6-P. After incubation, the relative activity of each radiopharmaceutical associated with the bacterial cells was calculated (Figure 2a).

The *S. aureus* RN6390 parent and *S. aureus* RN6390 *ΔuhpT* mutant strains demonstrated comparable uptake of [^18^F]FDG. However, only *S. aureus* RN6390 was able to transport and accumulate [^18^F]FDG-6-P, indicating that [^18^F]FDG-6-P was a specific substrate for the UHPT. In addition, the amount of [^18^F]FDG-6-P activity associated with the parent strain was significantly elevated compared with [^18^F]FDG (*P* = 0.0015).

In order for [^18^F]FDG-6-P to be a potential translational tool for the specific diagnosis of bacterial infections, it should not be transported into mammalian cells. Consequently, several mammalian cell lines of both human (HL60, Jurkat, AGS and THP1) and murine origins (HIB-1B and 3 T3-L1) were incubated with [^18^F]FDG or [^18^F]FDG-6-P, and the relative activity associated with the cells was determined (Figure 2b). For each cell line, significantly more [^18^F]FDG was associated with the cells than [^18^F]FDG-6-P (HL60 *P* = 0.0066; Jurkat *P* = 0.0089; AGS *P* = 0.0218; THP1 *P* = 0.0002; HIB-1B *P* = 0.0004; 3 T3-L1 *P* < 0.0001). The relative amount of [^18^F]FDG-6-P uptake in the mammalian cell lines was comparable with that of the UHPT-deficient staphylococcal mutant, indicating there was no specific mammalian cell transport system for the phosphorylated sugar.

### Biodistribution of [^18^F]FDG-6-P was similar to [^18^F]FDG

**Figure 3:**
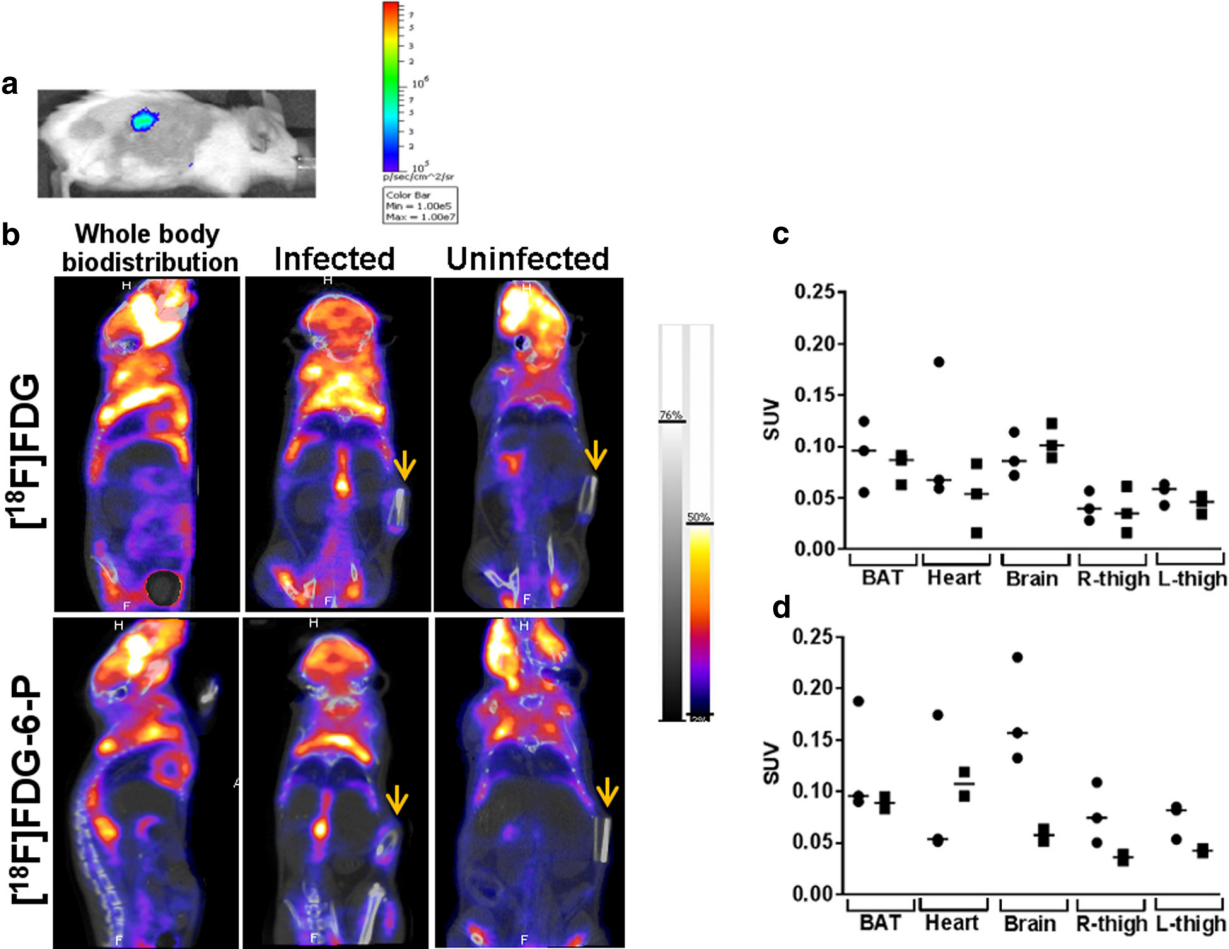
***In vivo*** **biodistribution of [** ^**18**^ **F]FDG-6-P and [** ^**18**^ **F]FDG. (a)** Representative optical image confirming the presence of BL catheter-associated *S. aureus* on the day of the nanoScan PET-CT scan. **(b)** Whole body biodistributions from nanoPET-CT scans of mice with sc implanted catheters inoculated with or without *S. aureus* 1 h after ip injection of approximately 10 MBq [^18^F]FDG or [^18^F]FDG-6-P; catheters are shown with arrows. SUVs were calculated from 3D ROIs drawn for each mouse from the whole-body nanoScan PET-CT images for **(c)** infected ([^18^F]FDG *n* = 3; [^18^F]FDG-6-P *n* = 3) and **(d)** uninfected mice ([^18^F]FDG *n* = 3; [^18^F]FDG-6-P *n* = 2. Black circle, [^18^F]FDG; black square, [^18^F]FDG-6-P). Bars on graph show median. BAT, brown adipose tissue.

Infections were confirmed by BL imaging of all mice (Figure 3a). Whole body nanoScan PET-CT imaging of mice with implanted catheters (infected and uninfected) 1 h post injection of approximately 10 MBq [^18^F]FDG-6-P was performed (administered doses ranged between 8 and 11 MBq). The biodistribution of [^18^F]FDG-6-P was shown to be visually comparable with that of [^18^F]FDG, which had been similarly administered by ip injection into a secondary cohort of mice (administered doses ranged between 8 and 11 MBq). No uptake of [^18^F]FDG-6-P into host tissues was anticipated; however, as for the [^18^F]FDG biodistribution, [^18^F]FDG-6-P activity was captured from the leg muscles, heart, brown adipose tissue (BAT) and brain (Figure 3b), as well as the infected catheter (Figure 3b, yellow arrows). The biodistribution of [^18^F]FDG and [^18^F]FDG-6-P was further quantified by calculating SUVs for the following organs: BAT, heart, brain, right thigh and left thigh. These were calculated for *S. aureus*-infected mice (Figure 3c, [^18^F]FDG *n* = 3 and [^18^F]FDG-6-P *n* = 3) and uninfected mice (Figure 3d, [^18^F]FDG *n* = 3 and [^18^F]FDG-6-P *n* = 2). The SUV values for specific organs varied per mouse; however, the trend demonstrated that infection did not greatly affect the whole-body biodistribution of either of the radiopharmaceuticals and that [^18^F]FDG-6-P biodistribution was not significantly different to that of [^18^F]FDG.

### [^18^F]FDG-6-P was not dephosphorylated in the blood

**Figure 4:**
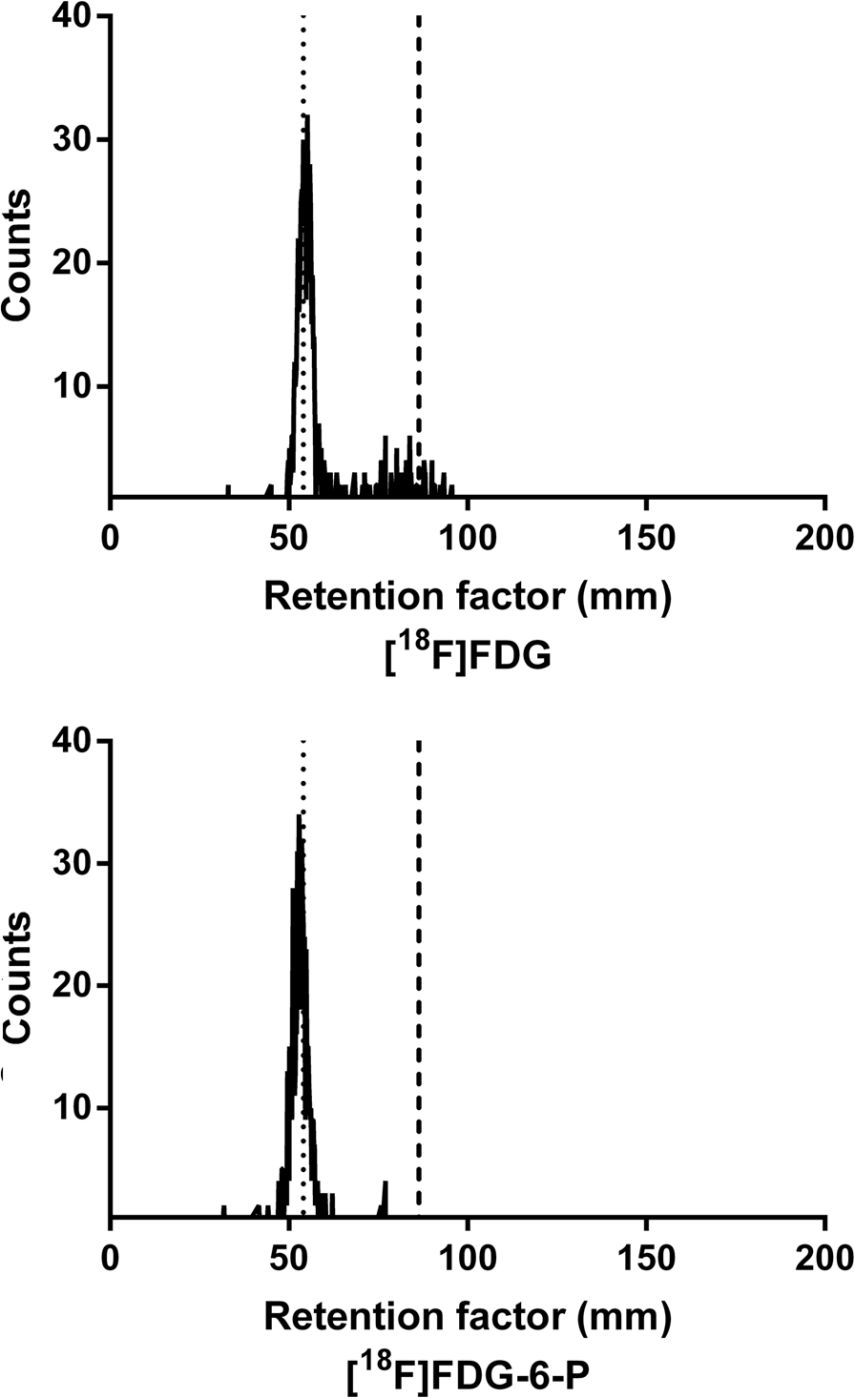
**Stability of [** ^**18**^ **F]FDG-6-P in the blood.** Blood extracted from mice was incubated with [^18^F]FDG or [^18^F]FDG-6-P for 1 h. The blood was analysed by iTLC to determine whether any additional peaks indicating dephosphorylation of [^18^F]FDG-6-P. The dotted line shows the peak for the [^18^F]FDG-6-P standard, and the dashed line shows the peak for the [^18^F]FDG standards which were not incubated with blood.

The *in vivo* biodistribution of [^18^F]FDG-6-P was highly unexpected. In order to explore whether the [^18^F]FDG-6-P was being dephosphorylated to produce [^18^F]FDG during systemic circulation, samples of mouse blood were collected and incubated with either [^18^F]FDG or [^18^F]FDG-6-P *ex vivo* for 1 h. The blood was analysed by iTLC to examine the peak profiles for each of the radiopharmaceuticals (Figure 4). As anticipated, the blood incubated with [^18^F]FDG showed two defined peaks; a large peak consistent with intracellular [^18^F]FDG-6-P (which arises from phosphorylation of [^18^F]FDG as it passes through glucose transporters into the cell) and a smaller [^18^F]FDG peak (indicating that some [^18^F]FDG had not yet been transported into the cells). The blood incubated with [^18^F]FDG-6-P had only one defined peak, consistent with the [^18^F]FDG-6-P standard which had not been incubated with blood, indicating that [^18^F]FDG-6-P was not being dephosphorylated to [^18^F]FDG.

### [^18^F]FDG-6-P provides a higher signal-to-noise ratio than [^18^F]FDG at the infection site

**Figure 5:**
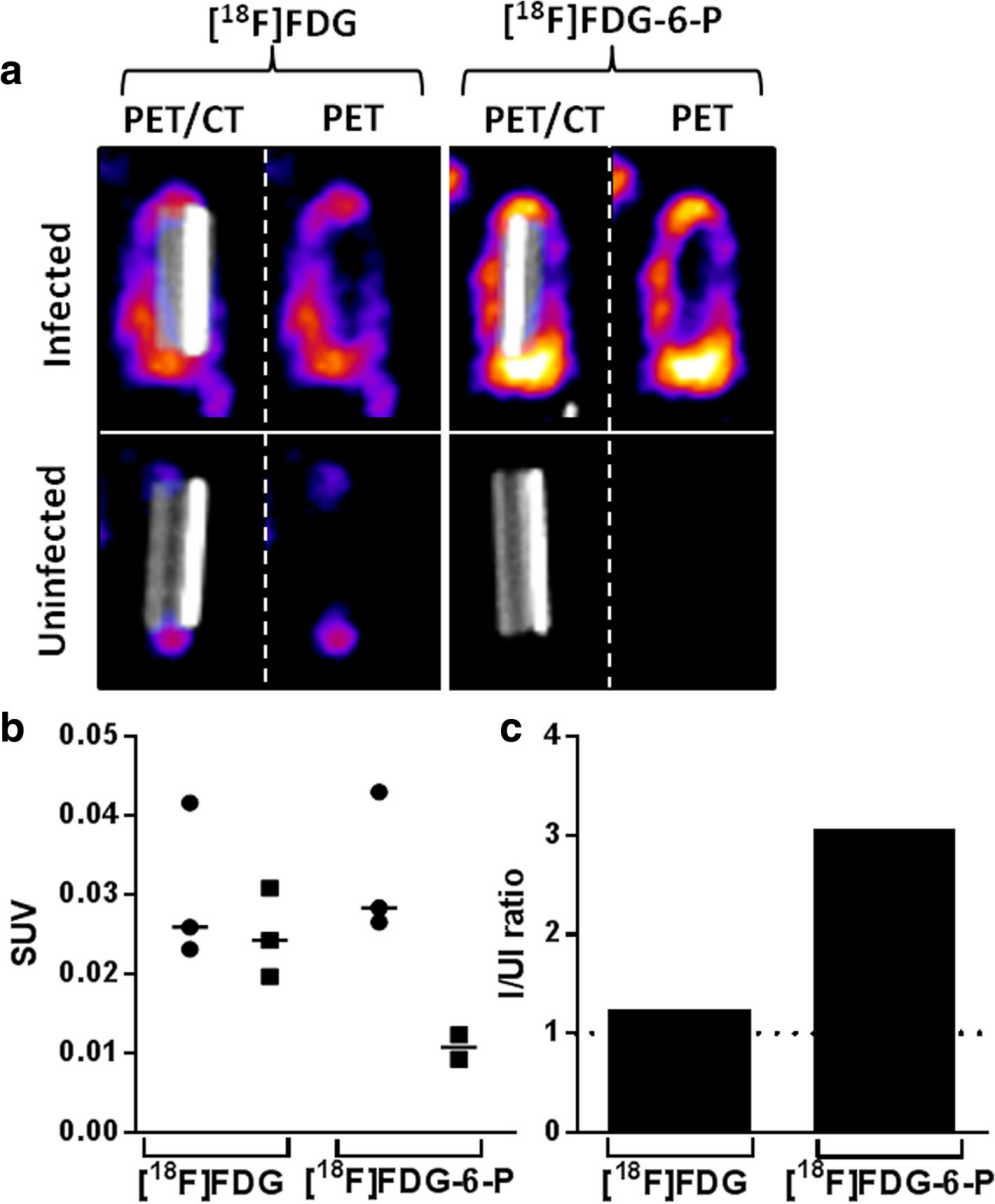
**Accumulation of [** ^**18**^ **F]FDG and [** ^**18**^ **F]FDG-6-P at the catheter infection site.** Mice with *S. aureus* infections (or uninfected control mice) were injected with approximately 10 MBq [^18^F]FDG (*n* = 3 infected, *n* = 3 uninfected) or approximately 10 MBq [^18^F]FDG-6-P (*n* = 3 infected, *n* = 2 uninfected) 1 h prior to nanoScan PET-CT imaging. **(a)** Representative 3D colour map of catheter regions cropped from whole-body nanoScan PET-CT images. Images are shown with and without CT. **(b)** SUV values were calculated for *S. aureus-*infected (black circle) and uninfected mice (black square). Mann-Whitney *U* tests confirmed that there were no significant differences in SUVs between infected and uninfected mice injected with either [^18^F]FDG (*P* = 0.7619) or [^18^F]FDG-6-P (*P* = 0.0556). Bars on the graph show median SUVs for each group (*n* = 3 for [^18^F]FDG infected and *n* = 3 for uninfected mice; *n* = 3 for [^18^F]FDG-6-P infected mice and *n* = 2 uninfected mice). **(c)** The infected (I) to uninfected (UI) ratio (I/UI) for the catheter sites of mice injected with [^18^F]FDG and [^18^F]FDG-6-P was calculated by dividing the mean infected catheter SUV by the mean uninfected catheter SUV for each cohort of mice.

Despite the unexpected whole-body biodistribution of [^18^F]FDG-6-P *in vivo*, the accumulation of [^18^F]FDG-6-P and [^18^F]FDG at the site of bacterial catheter foreign body infections was characterised. After image reconstruction of data captured from the nanoScan PET-CT scans (Figure 3b), the catheter regions were cropped from the whole body image and scales normalised based on injected activity in order to visualise activity associated with the catheter (Figure 5a). Visually, [^18^F]FDG was present at the site of both infected and uninfected catheters and as expected, [^18^F]FDG accumulated to a higher concentration when bacteria were present. The activity present at either end of the uninfected catheter could indicate inflammation at these positions where the rough, cut surface of the catheter was in contact with the tissue. For the infected mice, it was apparent that the majority of the activity was associated on the outside of the catheter and the surrounding tissue, rather than inside the lumen of the catheter where a large bacterial population was expected. These data suggest that the radiopharmaceutical may not be reaching the majority of bacterial cells.

A similar accumulation pattern was observed for infected mice injected with [^18^F]FDG-6-P (Figure 5a). However, differences in accumulation between the two radiopharmaceuticals were visualised at the catheter site of uninfected mice. Unlike [^18^F]FDG, [^18^F]FDG-6-P did not accumulate at the sites of sterile inflammation at the ends of the catheter, suggesting that [^18^F]FDG-6-P was not accumulating within the inflammatory cells.

The accumulation of [^18^F]FDG and [^18^F]FDG-6-P at the site of the catheter was further quantified by calculating the SUV of the activity around each of the catheters. The SUVs demonstrated that [^18^F]FDG was a poor tool for identifying bacterial infection due to high background signal in uninfected controls (Figure 5b); the trend in accumulation was only marginally increased for infected vs. uninfected mice. The SUVs for [^18^F]FDG-6-P injected mice (Figure 5b) demonstrated a lower background signal in uninfected mice compared to infected mice and compared with [^18^F]FDG control mice. However, the decrease was not significant.

Infected to uninfected (I/UI) ratios were calculated for the catheter site based on the mean SUV value for each group (Figure 5c). For each graph, a value of 1 would show equal SUVs for infected and uninfected mice. The I/UI ratio for [^18^F]FDG-6-P infected mice was more than double that of the [^18^F]FDG injected cohort, indicating a clear difference in accumulation of the radiopharmaceuticals at the sites of infection compared with inflammation.

## Discussion

The aim of this study was to address the ever-increasing need for better, bacteria-specific diagnostic radiopharmaceuticals for translation into the clinical setting. Here we have exploited an alternative sugar transporter unique to and highly conserved in several bacteria, thereby negating the current issues with targeting host inflammatory cells and the high non-specific background currently observed with [^18^F]FDG imaging. During the preparation of this manuscript, Gowrishankar [34] and Weinstein [35] reported the exploitation of bacteria-specific sugar transporters for maltose and sorbitol using 6-[^18^ F]-fluoromaltose [34] and [^18^F]-fluorodeoxysorbitol ([^18^F]FDS) respectively for pre-clinical imaging. [^18^F]FDS can be prepared from [^18^F]FDG, the most widely available PET imaging radiopharmaceutical and therefore [^18^F]FDS could potentially be used in any clinic with access to [^18^F]FDG. However, [^18^F]FDS is specific for the Gram-negative *Enterobacteriaceae* and so cannot be used for imaging *S. aureus* infections. The development of an imaging agent to detect *S. aureus* infections is therefore urgently required. As stated within the introduction, MRSA strains are the most widely isolated multi-antibiotic resistant bacteria found within European hospitals [20,21]. Moreover, *S. aureus* is particularly problematic and difficult to eradicate because of its ability to cause chronic infections through biofilm formation on indwelling medical devices such as catheters, pacemakers and prosthetic joints [36,37]. Furthermore, due to the expanding ageing population, there is an ever-increasing demand for such devices, placing more patients at risk of contacting staphylococcal infections [38].

To create a radiopharmaceutical with translatable potential for specifically identifying bacterial infections, including those caused by *S. aureus*, the UHPT was selected as a target [30]. The UHPT is conserved amongst several bacterial strains, including *Staphylococcus*, *Escherichia*, *Shigella* and *Enterobacter*, and is able to transport a number of hexose-6-phosphate sugars. The UHPT transporter is highly expressed by *S. aureus* and due to clinical demand for tools to diagnose this pathogen, it was selected for use in this study.

Due to its accepted clinical use and widespread availability, we chose to modify [^18^F]FDG by converting it to the corresponding phosphate analogue, [^18^F]FDG-6-P. The latter was confirmed as a substrate for the bacterial UHPT in *in vitro* accumulation assays in comparison with [^18^F]FDG. *S. aureus* RN6390 and the isogenic UHPT mutant strain accumulated [^18^F]FDG to the same extent, although the level of [^18^F]FDG-6-P in the parental cells was significantly higher. This was consistent with the elevated growth rates observed when *S. aureus* RN6390 was supplemented with glucose-6-phosphate instead of glucose (data not shown), suggesting that the UHPT was active and could allow even higher levels of [^18^F]FDG-6-P to accumulate at the site of infection compared with [^18^F]FDG, resulting in an elevated signal-to background ratio. Accumulation assays with [^18^F]FDG and [^18^F]FDG-6-P and various mammalian cell lines *in vitro* were performed to confirm that eukaryotic cells did not transport [^18^F]FDG-6-P. It is important to note that both murine and human-derived cell lines were used for the *in vitro* assays and no differences in radiopharmaceutical accumulation were observed. Therefore, it was anticipated that [^18^F]FDG-6-P biodistribution and accumulation in both mice and humans would be similar.

Pre-clinical *in vivo* infection studies with [^18^F]FDG-6-P were therefore performed. A foreign body model of infection with *S. aureus* was used to replicate clinical situations where biofilm formation results in chronic infections that are refractory to antibiotic therapy [36,39,40]. For these studies, the mice were not fasted, warmed or maintained under anaesthetics between radiopharmaceutical administration and imaging as is suggested for optimal [^18^F]FDG imaging [41,42] because it was anticipated that non-specific background from [^18^F]FDG-6-P after clearance from the blood pool would be minimal. Mice injected with [^18^F]FDG were prepared identically to those injected with [^18^F]FDG-6-P to enable direct comparisons. All mice were imaged optically to confirm equivalent infection levels prior to [^18^F]FDG-6-P or [^18^F]FDG administration.

Unexpectedly, very high levels of background signal from mice injected with [^18^F]FDG-6-P were observed for both infected and uninfected mice. The whole-body biodistribution of [^18^F]FDG and [^18^F]FDG-6-P was visually similar, and this was confirmed when SUVs were calculated from ROIs of the brown adipose tissue, heart, brain and right and left thighs. The first potential explanation for this finding was that the [^18^F]FDG-6-P was either unstable or was specifically dephosphorylated within the systemic circulation in the mouse, resulting in the release of free [^18^F]FDG which could then be transported into mammalian cells by GLUT transporters. However, glucose-6-phosphatase distribution in mice has previously been studied with *ex vivo* whole-body autoradiography [43]. This study revealed that high levels of the enzyme were present in the liver, kidney, intestine and skeletal system, but not in the circulatory system. The stability of [^18^F]FDG-6-P in the blood was also confirmed by *ex vivo* incubation and analysis by iTLC.

Despite the accepted understanding that mammalian cells do not transport phosphorylated sugars, there have been few reports in the literature. To our knowledge, there is only one published study investigating glucose-6-phosphate uptake in mammalian cell lines [44]. However, the authors speculated that the apparent growth of the cells incubated with glucose-6-phosphate may be a result of proteins in their tissue culture medium dephosphorylating the glucose-6-phosphate, releasing glucose for transport and subsequent metabolism. Unfortunately, no further studies to determine whether mammals are able to transport phosphorylated sugars have been reported.

Critically, despite the similarity in the whole-body biodistribution of [^18^F]FDG-6-P and [^18^F]FDG, differences between the two radiopharmaceuticals were observed at the site of the catheter implant for uninfected mice. [^18^F]FDG but not [^18^F]FDG-6-P accumulated at the ends of the catheters which are likely to be sites of sterile inflammation. It can be assumed that similar levels of inflammation were present at the ends of the catheters as all catheters were prepared and implanted in the same way. This suggests that the two radiopharmaceuticals behave differently and that [^18^F]FDG-6-P was not present as [^18^F]FDG. This raises the prospect that the observed uptake of [^18^F]FDG-6-P into the other organs may be a real process and so warrants further investigation to determine whether this phenomenon extends to man. Importantly, the reduced [^18^F]FDG-6-P activity at the catheter site of uninfected mice in this proof of concept study demonstrated that the [^18^F]FDG-6-P activity around the infected catheter is associated specifically with *S. aureus* and not inflammatory cells; however, due to the small sample size, it is not possible to determine statistical significance.

## Conclusions

Based on our *in vivo* studies, it is clear that [^18^F]FDG-6-P did not behave as expected within the mouse model; however, the radioactivity at the infection site was elevated more than twofold for infected mice compared with uninfected controls injected with [^18^F]FDG-6-P and compared with [^18^F]FDG. This indicated that [^18^F]FDG-6-P did not accumulate within the inflammatory cells and so this could conceivably provide a means to distinguish between sites of sterile inflammation and *S. aureus* infection, an important limitation of [^18^F]FDG imaging [45]. In addition, the UHPT is also able to transport other hexose phosphates, such as mannose-6-phosphate and fructose-6-phosphate (data not shown). We propose therefore that fluorinating these sugars with [^18^F] may enable the specific targeting of the UHPT, reducing background uptake and provide a more favourable biodistribution for *in vivo* imaging.

## Abbreviations

BAT: brown adipose tissue
BL: bioluminescent
CA: community-acquired
CFU: colony-forming units
cpm: counts per minute
DEX: dexamethasone
FCS: foetal calf serum
FDG: fluorodeoxyglucose
FDGP: fluorodeoxyglucose-6-phosphate
FOV: field of view
IBMX: 3-isobutyl-L-methylxanthine
I/UI: infected/uninfected ratio
iTLC: instant thin layer chromatography
HA: hospital-acquired
ip: intraperitoneal
MDR: multi-drug resistant
MRSA: methicillin-resistant *Staphylococcus aureus*
P/S: penicillin/streptomycin
Rf: retention factor
ROI: region of interest
RP-HPLC: reverse-phase high-performance liquid chromatography
*S. aureus*: *Staphylococcus aureus*
sc: subcutaneous
SEM: standard error of the mean
SUV: standardised uptake value
T3: triiodo-L-thyronine
TSB: tryptic soy broth
UHPT: universal hexose phosphate transporter
